# De novo mutations in *SOD1* are a cause of ALS

**DOI:** 10.1136/jnnp-2021-327520

**Published:** 2021-09-13

**Authors:** Kathrin Müller, Ki-Wook Oh, Angelica Nordin, Sudhan Panthi, Seung Hyun Kim, Frida Nordin, Axel Freischmidt, Albert C Ludolph, Chang Seok Ki, Karin Forsberg, Jochen Weishaupt, Young-Eun Kim, Peter Munch Andersen

**Affiliations:** 1 Department of Neurology, Ulm University, Ulm, Germany; 2 Department of Neurology, Hanyang University Seoul Hospital, Seongdong-gu, Seoul, Republic of Korea; 3 Cell Therapy Center, College of Medicine, Hanyang University, Seoul, Republic of Korea; 4 Clinical Science, Neurosciences, Umeå University, Umeå, Sweden; 5 Genome Research Centre, GC Genome, Yongin, Republic of Korea; 6 Medical Biosciences, Umeå University, Umeå, Sweden; 7 Department for Neurodegeneration, Universitätsmedizin Mannheim, Mannheim, Germany; 8 Department of Laboratory Medicine, Hanyang University College of Medicine, Seoul, Republic of Korea

**Keywords:** ALS, neurogenetics, motor neuron disease

## Abstract

**Objective:**

The only identified cause of amyotrophic lateral sclerosis (ALS) are mutations in a number of genes found in familial cases but also in sporadic cases. De novo mutations occurring in a parental gonadal cell, in the zygote or postzygotic during embryonal development can result in an apparently sporadic/isolated case of ALS later in life. We searched for de novo mutations in *SOD1* as a cause of ALS.

**Methods:**

We analysed peripheral-blood exome, genome and Sanger sequencing to identify deleterious mutations in *SOD1* in 4000 ALS patients from Germany, South Korea and Sweden. Parental kinship was confirmed using highly polymorphic microsatellite markers across the genome. Medical genealogical and clinical data were reviewed and compared with the literature.

**Results:**

We identified four sporadic ALS cases with de novo mutations in *SOD1*. They aggregate in hot-spot codons earlier found mutated in familial cases. Their phenotypes match closely what has earlier been reported in familial cases with pathogenic mutations in *SOD1*. We also encountered familial cases where de novo mutational events in recent generations may have been involved.

**Conclusions:**

De novo mutations are a cause of sporadic ALS and may also be underpinning smaller families with few affected ALS cases. It was not possible to ascertain if the origin of the de novo mutations was parental germline, zygotic or postzygotic during embryonal development. All ALS patients should be offered genetic counselling and genetic screening, the challenges of variant interpretation do not outweigh the potential benefits including earlier confirmed diagnosis and possible bespoken therapy.

## Introduction

Mutations in more than 40 genes have been linked to the pathogenesis of amyotrophic lateral sclerosis (ALS) and some also to the genetically linked frontotemporal dementia (FTD). The heritability in ALS is estimated to be 40%–53%, but only 5%–13% of patients report a positive family history for ALS.^1^ Mutations in the five exons encoding Superoxide-Dismutase 1 (CuZn-SOD; *SOD1*) is one of the more frequently encountered causes of hereditary ALS (hALS).^2^ Since the first report in 1993, coding mutations in *SOD1* have been found in 8%–23% of patients diagnosed with familial ALS (fALS) but also in 1%–4% of patients without an overt family history (denoted sporadic or simplex ALS, sALS).^3^ The occurrence of coding mutations in sALS has been explained by incomplete family history, the child developing ALS before the mutation-transmitting parent also develops ALS, illegitimacy, misdiagnosis in relatives who had ALS, pleitropism for other phenotypes (eg, six *SOD1* mutations have been associated with FTD)^3 4^ disguising the predisposition for hALS, reduced disease penetrance and recessive inheritance.^3^ While the vast majority of mutations have been found in heterozygous form, the prevalent *SOD1* mutation p.Asp91Ala is inherited as a recessive trait.^5 6^ These hereditary cases may present as seemingly sporadic disease. The discovery of inclusions staining positive for unfolded SOD1 exclusively in the motor neurons and glia cells in autopsied sALS patients without a *SOD1* mutation (analysed in DNA from blood leucocytes) suggest that an unfolded SOD1 protein may be playing a broader role in causing ALS.^7–9^ Hypothetically, de novo mutations could explain a proportion of sALS patients. While mosaicism for somatic *SOD1* mutations is an attractive hypothesis to explain sALS, it has never been proven to exist.^10^ Here, we describe four de novo variants in *SOD1* in sALS cases. The finding raises questions about the mutation frequency in *SOD1*, whether de novo mutations contribute to sALS and to smaller fALS pedigrees, and if genetic counselling and screening should be performed routinely in all ALS cases.

## Materials and methods

Approved by the medical ethical committees in alignment with the Declaration of Helsinki (WMA, 1964), as part of longitudinal nationwide projects in Germany, South Korea and Sweden, newly diagnosed ALS patients are invited to donate blood sample for genetic research into the aetiology of ALS. With written informed consent, a genealogical history is obtained and venous blood collected into EDTA-containing vacuum tubes. Leucocyte DNA is extracted using standard procedures. Patient samples are screened for a panel of genes, in many whole-exome sequencing or whole-genome sequencing are also performed (detailed information available on request). Variants are called using GATK software. We filter out variants with allele frequencies>0.01 based on public genome variant databases (eg, the genome aggregation database (gnomAD, https://gnomad.broadinstitute.org), 1000 genomes (http://www.1000genomes.org/), dbSNP (http://www.ncbi.nlm.nih.gov/SNP/), the Korean Reference Genome Database (KRGDB, http://coda.nih.go.kr/coda/KRGDB/index.jsp) and the Swedish variant data set (https://swegen-exac.nbis.se)). *SOD1* is studied by bidirectional Sanger sequencing of all five exons and 30–50 bp of adjacent introns as described.^11^ Sequence variations are compared with reference sequence for *SOD1* (NM_000454.4). The *C9orf72HRE* was excluded by RP-PCR and fragment length analysis. When also erythrocytes were available, the SOD1 enzymatic activity in erythrocytes were assayed.^11^ The variants were assessed for pathogenicity using the American College of Medical Genetics and Genomics (ACMG) criteria.^12^

Paternity and maternity analysis: following the discovery of *SOD1* mutations in the four sALS cases and after genetic counselling, medical genealogical studies were performed in the families for at least three generations. Particular attention was paid for possible misdiagnosed earlier cases of ALS or FTD (none were found). All four families were informative. At the DNA level, paternity and maternity was studied using 15 highly polymorphic short tandem repeats (STR) loci and a sex identification marker, amelogenin, using the Promega PowerPlex16 System (PPP16) (Promega Corporation, Madison, WI). Analysis were done following the manufacturer’s instructions.

The patients were diagnosed in accordance with established guidelines^13^ and were examined at least once in a university clinic specialised in ALS care.

## Results

This report focuses on the four patients who were found to carry mutations in *SOD1* that could be confirmed to be of de novo origin. To find these, we screened over 4000 ALS cases (Germany 1400, Korea 1100, Sweden 1600). Mutations were discovered in other patients diagnosed with sALS but either genealogical investigations revealed relatives with ALS/FTD, analysis showed one of the parents to be an asymptomatic mutation carrier or the pedigrees were non-informative (ie, small families, DNA samples were not available from one or both parents, the parents died young of other causes or were lost to follow-up, essential records could not be retrieved). Manuscripts describing the overall population cohorts are in preparation.

### Clinical depictions and genetic results

A summary of the participants is presented in online supplemental table 1.

10.1136/jnnp-2021-327520.supp1 Supplementary data

Patient A: at the age of 22, 5 years this German woman experienced muscle cramps, progressive paresis and muscle wasting beginning in one leg and spreading to the other leg without sensory, autonomic or cognitive symptoms. A myopathy was initially suspected but EMG revealed acute and chronic signs of denervation mostly in the legs but also slightly in the upper limbs. Transcortical magnetic evoked potential (MEP) examination was pathological with delayed central conductance time to both lower limbs but not to the upper limbs. Peripheral nerve conduction studies were normal. The medical history was unremarkable except for a voluntarily weight loss of ≈10 kg in the years before appearance of muscle symptoms. Both the parents have diabetes mellitus type 2, but there is no family history of a neuromuscular disease (NMD) or FTD-like condition. The patient eventually received a diagnosis of sALS. The disease has developed slowly, symptoms and signs of upper motor neuron (UMN) and lower motor neuron (LMN) damage only appearing in the upper limbs 3 years after onset in the lower limbs. Nine years after onset of paresis, the patient is alive and still show no bulbar symptoms.

Genetic analysis: because of her young age and the difficulties in setting a diagnosis, initially a neuromuscular panel of 443 genes was screened. This revealed a novel heterozygous c.112G>C (p.Gly38Arg) mutation that was confirmed by Sanger sequencing in two other laboratories. Both parents were without this mutation but parental DNA analysis confirmed them to be the patient’s biological parents (online supplemental figure 1a and online supplemental table 2a). Examinations of the parents were normal. No other possible pathogenic variant for the phenotype in Patient A was found. A c.112G>A also resulting in a p.Gly38Arg exchange was one of the original *SOD1* mutations found to cause ALS following linkage analysis of fALS pedigrees.^2^ This mutation has been reported in fALS patients in Germany, Spain, Taiwan, Turkey and the USA and overexpression in a transgenic mouse model results in a murine MND.^14^ The delay in central conduction time on MEP stimulation observed in Patient A has earlier been observed in ALS patients with *SOD1* mutations.^15^ All evidence supports that a p.Gly38Arg substitution in *SOD1* can cause ALS (online supplemental table 1).

10.1136/jnnp-2021-327520.supp2 Supplementary data

10.1136/jnnp-2021-327520.supp3 Supplementary data

Patient B: 4 years before developing paresis, this Korean woman had experienced troublesome muscle cramps and a tingling sensation in the right leg. These symptoms disappeared a year before overt paresis and atrophy appeared in the same leg. The paresis has slowly generalised to now all four extremities with signs of UMN and LMN involvement also in the cranio-bulbar and cervical region with increased jaw jerk and tongue fasciculations but without dysarthria or dysphagia. Sensory functions are normal. The patient was diagnosed with sALS and is alive 41 months after onset without needing non-invasive ventilation or a gastrostomy. Overall disease progression rate is slow at 0.46 (ALSFRS-R 48-29/41 months).

The patient is the oldest of three siblings in an informative family: the parents are in their late 60s, the father suffers from arterial hypertension and diabetes mellitus, while the mother is healthy. The parents have multiple siblings and they and the grandparents are without a history of ALS or FTD. A nephew has Myotonic dystrophy.

DNA analysis revealed Patient B to be heterozygous for a c.268G>A (p.Ala90Thr) which was not found in the parents (online supplemental figure 1b). Paternity and maternity were confirmed using PPP16 (online supplemental table 2b). A similar c.268G>A (p.Ala90Thr) has earlier been reported in a US-fALS family of Hispanic descent with a somewhat similar phenotype. In this family, the proband had leg-onset of slowly progressing paresis and atrophy beginning at age 26,5 years with joint and back pain and intermittent hot sensations in both legs as atypical features.^16^ Such symptoms have not been reported by Patient B.

The c.268G>A (p.Ala90Thr) fulfils the ACMG criteria for being a pathogenic variant based on its absence in control populations in databases, deleterious results in multiple lines of in-silico analysis, a de novo mutation and previously reported in fALS (online supplemental table 1).

Patient C: at age 42 years, this Swedish woman developed progressive paresis and wasting beginning in the left hand spreading to the entire left arm, then to the left leg, the right arm and leg. The patient had no sensory signs but experienced troublesome diffuse pain symptoms in the left shoulder region prior to onset of paresis in the hand. This delayed the diagnosis. Clinical examinations, imaging, neurophysiological and blood and CSF workup were compatible with ALS, no other cause for her symptoms was found. Having no family history for ALS or FTD, she received a diagnosis of sALS 26,5 months after onset of paresis. She eventually developed bulbar symptoms and died from respiratory failure 50,3 months after onset.

DNA analysis revealed a heterozygous c.272A>T p.Asp91Val mutation in the proband but not in her parents (collected and analysed twice, online supplemental figure 1c) or siblings. Their kinship was confirmed with high probability by PPP16 analysis (online supplemental table 2c). No other possible pathogenic mutation for ALS was found. The mutation has earlier been reported in a small 2-generation Japanese fALS family.^17^ The collective evidence classify the c.272A>T p.Asp91Val as pathogenic for ALS (online supplemental table 1).

Patient D: this patient first presented at age 30 with diffuse pain syndrome in the shoulder region followed by onset of paresis and muscle wasting in his left arm. A neuralgic shoulder amyotrophy was initially suspected but treatment with corticosteroids had no effect. Within 5 months, the paresis and wasting had spread also to the right arm and left leg. In the lower extremities, the onset of manifest paresis was preceded by a period of troublesome pain sensation requiring treatment with analgesics. An examination 10 months after onset showed severe muscle paresis and atrophy and reduced cough strength. There was no dysarthria, dysphagia or difficulty holding the head. Deep-tendon reflexes were reduced in the upper extremities and the left leg. EMG analysis revealed acute and chronic denervation in the limbs. Routine blood, CSF analysis and MRI of head and spine were normal. Fifteen months after onset, he had lost his ability to walk and dysarthria and dysphagia appeared. Soon after, non-invasive ventilation treatment was initiated but was unsuccessful and he was placed on permanent invasive ventilation. He now lives in a palliative care facility in an almost lock-in state 38 months after onset.

**Figure 1:**
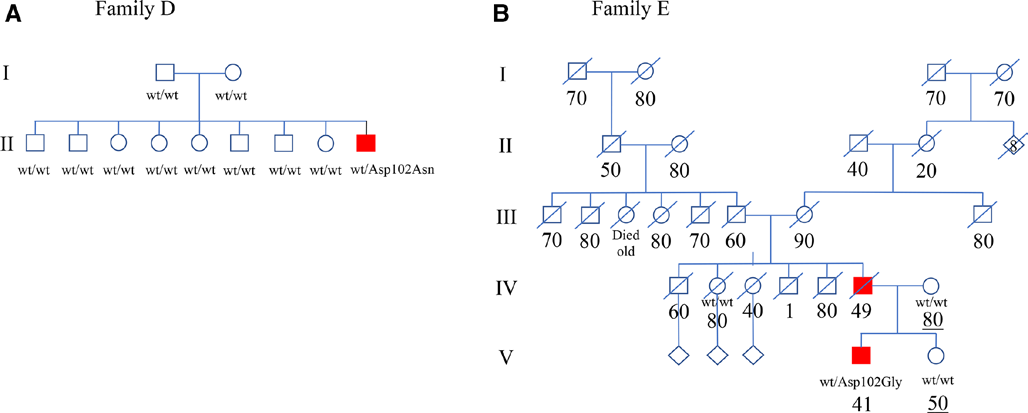
(A) Pedigree of Family D where only the youngest member in generation II has developed ALS. He tested positive for heterozygosity for the p.Asp102Asn *SOD1* mutation. The parents are in their late 60s and early 70s, respectively. Open symbols, unaffected family members. (B) Abbreviated pedigree of Family E. The family comes from an area where extensive ALS research has been performed for decades but only two the father (IV-6) and son (V-4) with ALS could be found in the extended family. V-4 tested positive for heterozygosity for the p.Asp102Gly *SOD1* mutation. Ages at death (per decade) are listed below each individual. Changes have been made to the pedigree for anonymisation purposes. ALS, amyotrophic lateral sclerosis; Wt, wild type SOD1 allele.

The patient comes from a large informative Kurdish family without a history of NMD or FTD (figure 1). Genetic analysis revealed a heterozygous variant c.304G>A (p.Asp102Asn) in *SOD1* which was absent in his parents and eight older siblings (online supplemental figure 1d). The PPP16 result supports kinship (online supplemental table 2d). The same variant was first reported in a sALS patient of Pakistani origin living in England^18^ and later in fALS families from Pakistan and Belarus.^19 20^ Three other mutations have been reported in codon 102 in ALS patients with an aggressive phenotype similar to this case. The evidence classify the c.304G>A (p.Asp102Asn) as being pathogenic for ALS (online supplemental table 1).

## Discussion

Some 220 mutations that is predicted to alter the SOD1 amino acid sequence have been found since 1993 (https://alsod.ac.uk).^3 21^ For only about a quarter of these have causation for ALS been proved by cosegregation in large pedigrees with a significant LOD score, by statistical–epidemiological means or by overexpression in a transgenic rodent model that develops an ALS-like disease. A further quarter of mutations are in the same or adjacent codons to mutations with proven pathogenicity and a ‘hot-spot’ argument for pathogenicity can be presented.^22^ Conversely, after 28 years of screening many thousands of ALS patients for *SOD1* mutations, there are obvious cold-spot codons in *SOD1* where few or no mutations have been reported in ALS (or controls). These includes codons 23 through 36 and the codons encoding the active-site channel. Thirty-one mutations interfere with intron-exon splicing, or introduces or deletes nucleotides and hence can be predicted to alter the length of the mutant protein. Many of these have been reported in single cases with a sALS diagnosis and do not fulfil basic causality criteria. Frequently, the parents had not been genotyped and it is unknown if they were asymptomatic mutation carriers, the biological parents of the reported patient or if the reported mutation was a de novo mutational event. Convincing data for pathogenicity showing that a 153 aa full-length SOD1 polypeptide is not necessary for causing ALS has been published only for a few non-sense mutations with a fALS diagnosis (eg, p.Lys128GlyfsTer6).^23 24^ The discovery of ALS-causing truncated mutants that are unable to form the native SOD1-structure and that patients with such mutations have the same phenotype and histopathology as patients caused by missense mutations, suggest that pathogenesis is initiated from the unfolded SOD1 polypeptide sequence. Denominators for fALS-causing mutations are either a reduction in the surface repulsive charge of the mutant protein making it more aggregation prone (wild-type SOD1 has a charge of −6), a reduction of the Cys58-Cys147 intrasubunit disulfide bridge and/or loss of binding the zinc cofactor. Loss of any of these will unfold the robust SOD1 monomer.^25^ Evidence suggests that mutant SOD1 cause ALS by an aquired cytotoxic function and not by loss of enzymatic activity.^5^ In transgenic mice ALS-models overexpressing *SOD1* mutations found in fALS, two conformational strains of SOD1-prions have been identified.^26^ One of these have been purified from spinal cord autopsy tissue of a fALS patient with the p.Lys128GlyfsTer6 *SOD1* mutation and elicit ALS disease when inoculated into the spinal cord of mice.^23^ It is not yet possible to assay for the putative SOD1-prion species in the CSF of patients nor has it been established in which cell types the prions are active in to cause disease. While the finding of a reduction in SOD1 enzymatic activity is indicative of the presence of a *SOD1* mutation, six fALS-causing mutations have preserved SOD1 activity.^5 11^ Hence, there is no non-genetic method yet available to determine whether a mutation in *SOD1* is pathogenic or not. The large number of mutations for a small gene and their unexplained uneven distribution across the molecule makes the interpretation of novel genetic test results difficult.

We here provide compelling evidence that de novo mutations in *SOD1* may be the cause of four simplex cases of sALS (online supplemental table 1). While the median onset of motor symptoms in sALS overall is ≈58–60 years, the median onset for fALS with high penetrant *SOD1* mutations is ≈47 years but for some mutations even lower.^22 27^ All four had spinal onset which is also more common among *SOD1* fALS cases than bulbar-onset.^22^ All four had UMN signs but LMN involvement was the dominant feature as it is in fALS-caused by *SOD1* mutations.^16 22^ The disease progressing rate varied greatly among the four cases but so is also the case for patients with different fALS mutations.^19^ A clinical feature was sensory symptoms (but not signs) often in the form of a pain syndrome preceding the onset of motor symptoms, resembling the pseudopolyneuritic variant of ALS (Patrikios’ Disease). These often delayed the ALS diagnosis but are in character no different from the sensory symptoms reported in *some* patients with the p.Ala5Val, p.Ala90Val, p.Asp91Ala and p.Gly128Arg *SOD1* mutations.^15 16 28 29^ In summary, the phenotypes observed closely matches what has been reported for fALS *SOD1*.

An important finding here is that three of the four mutations have earlier been reported in fALS cases in other ethnic populations. Using informative polymorphic markers proof of paternity and maternity was established with high confidence. This raises the hypothesis that there might be an increased propensity for ALS-causing mutational events in some codons of *SOD1*. While some fALS *SOD1* mutations are probably old mutational events —for example, all p.Asp91Ala *SOD1* have in a haplotype study been traced to a single ancestor ≈ 18 000 years ago^30^—the second most-frequent *SOD1* mutation globally p.Ala5Val appears to be on (at least) three different haplotypes suggesting that three mutational events have taken place through history to form the p.Ala5Val-SOD1 population we observe today.^31^ Supporting a mutational hot-spot theory is that while seven different mutations have been found in codon Ala5, only one has been found in codon Lys4 and two in Val6.^21^

Another intriguing observation is that some *SOD1*-fALS pedigrees are small, only having two or three affected members and ALS can often only be traced back 2–3 generations.^32^ This is in contrast to our fALS families associated with mutations in *C9orf72HRE* which frequently can be followed several generations back. An example is a two-generation father and son fALS family associated with heterozygosity for the p.Asp102Gly (Patient E, online supplemental table 1; figure 1b): the family originates in northern Sweden where we since the 1980s have performed extensive search to ascertain every possible case of ALS for a registry and where every diagnosed case since 1993 have been genotyped for ALS genes. This is the only family in any of the Nordic countries with this mutation. Speculatively, the affected father of Patient E may have been a de novo mutational event. The same p.Asp102Gly mutation has been found in England, Ireland and the USA and it involves the same codon Asp102 as our de novo case Patient D. A total of four mutations have been reported in codon 102 of which two have been found to cosegregate in large fALS pedigrees, while two others are in small fALS pedigrees.^19 21 33^ We propose that codons Ala5 and Asp102 may be hot spots for de novo mutations causing ALS.

Prior to this study, there has only been one reported finding of a de novo *SOD1* mutation in sALS, a young Irish man heterozygous for c.242A>G (p.His81Arg).^34^ While screening for this study, we surprisingly encountered the same c.242A>G (p.His81Arg) mutation in a Korean sALS patient but DNA analysis revealed the unaffected mother to carry the same mutation. His81 is critical for liganding the stabilising zinc ion and is evolutionarily highly conserved. Substituting His81 for Arg81 will result in a SOD1 subunit with increased propensity to unfold. Few mutations in this region have been reported and almost all are in patients with a diagnosis of sALS with onset before age 35 years.^22^

Corroborating that de novo mutational events may be a cause of sALS are 11 reports of de novo mutations in another ALS-causing gene *FUS*.^35^ Of these, the finding of the p.R496X *FUS* in an Italian sALS patient^36^ is of particular interest since the same mutation has been found to cosegregate with fALS in *small* families in Germany,^37^ Great Britain and Sweden, mirroring what we here report for sALS/fALS with *SOD1* mutations. Genomic de novo mutations have also been reported in single sALS cases in *ATXN2, SS18L1, CHRM1, ERBB4, VCP* and *RAPGEF2* genes raising the question: how frequent are de novo mutations as a cause of ALS?^38^

De novo mutations occurring in a parental gonadal cell or in the zygote will result in the mutant variant ending up in all cells in the foetus and may result in an apparently sporadic case of ALS later in life. Postzygotic somatic mutations will result in a mosaicism with mutant cells possibly ending up in the neuromuscular system and predisposing to ALS. If the somatic mutation occurs early postzygotic, mutant cells may end up also in the germline (and the new variant can potentially be transmitted to the next generation, ie, a fALS situation is created as we propose may have happened in Patient E’s family). If the de novo mutation occurs later, the germline will be spared but may still involve the stem cells forming the leucocyte lines and the new variant can be detected in DNA extracted from peripheral blood. A ‘late’ somatic mutation occurring in the developing ectoderm will not appear in leucocytes and result in a variant only existing in the neuromuscular system. The finding in chimeric mice^39^ that expression exclusively in motor neurons is not sufficient to cause motor neuron degeneration but mutant *SOD1* must also be expressed in adjacent non-neuronal cells, is relevant since it shows which cell lines that must be mutated to cause ALS. Data from chimeric mice suggest that expression of mutant SOD1 exclusively in interneurons, astrocytes and microglia is sufficient to cause ALS, ie, in cells that are mitotically active also in adults.^39^ Speculatively, a ‘late’ de novo mutation could occur within the spinal cord and cause true sALS and would not end up in the blood leucocytes used for DNA testing. In this study, when available, we also analysed SOD1 DNA in epidermis fibroblasts (ectodermal derived) and the SOD1 enzymatic activity in erythrocytes (mesodermal derived) (online supplemental table 1). The results were compatible with the genotype results from blood leucocytes and do not help us establishing when the four de novo mutational events occurred. For other diseases, a correlation has been observed between parental age and increased occurrence of germline mutations. The parents were all below 40 years of age when the four de novo cases were conceived. Examination of DNA chromatographs for their parents and siblings does not give a hint that a mosaicism scenario may be present (online supplemental figure 1a–d), but the sensitivity to detect a low-prevalent mosaicism is probably low.

The present finding of pathogenic de novo SOD1 mutations in sALS has ethical, clinical and research implications. The genomic revolution in medicine has reached lay people and as clinicians we are now frequently asked ‘what are my DNA results and *will my children be at risk*?’ expecting such analysis to have been performed. The findings of increasing number of sALS cases to have a genetic underpinning coupled with the promising results from new bespoken gene therapies^40^ make us revise our previous opinion.^13^ We now propose that all ALS patients should be offered genetic counselling and testing, or at least, with written informed consent blood should be collected and stored for future studies. Only the accessibility of blood-DNA from the parents of these four sALS cases made it possible to identify them as de novo mutational events.

## Data Availability

Data are available upon reasonable request. All data relevant to the study are included in the article or uploaded as supplementary information.
