# Supplementary material for: De novo mutations in SOD1 are a cause of ALS
Source: J Neurol Neurosurg Psychiatry. 2021 Sep 13;93(2):201–6. doi: 10.1136/jnnp-2021-327520 (PMC8784989; doi:10.1136/jnnp-2021-327520)
Supplement: Supplementary data [file jnnp-2021-327520supp001.pdf]

Supplementary Table 1: Demographics and clinical overview of the partcipants

	PATIENT				
	A	B	C	D	E
Origin	Germany	Korea	Sweden	Kurdistan	Sweden
Mutation	p.(Gly38Arg)<br>c.112G>C<br>GGA>CGA	p.(Ala90Thr)<br>c.268G>A<br>GCT>ACT	p.Asp91Val<br>c.272A>T<br>GAC>GTC	p.(Asp102Asn)<br>c.304G>A<br>GAT>AAT	p.Asp102Gly<br>c.305A>G<br>GAT>GGT
Mutation history report	Novel; A c.112G>A also resulting in p.G38R has been reported in fALS in Germany, Spain, Taiwan, Turkey, USA (ref. 2). Also expressed in tg mouse MND-model (ref. 14)	USA fALS of hispanic descent (ref. 16)	One small fALS in Japan (ref. 17)	Reported in a single sALS in the UK of Pakistani origin and 15 fALS cases in Belarus and Scotland (ref. 18, 19, 20)	fALS in England, Ireland, U.S.A. (ref. 19, 33)
"hot-spot" area of *SOD1* ?	yes	yes	yes	yes	yes
Charge change of mutant SOD1 compared to wild-type SOD1 (-6)	+1	neutral	+1	+1	+1
Sex	f	f	f	m	m
Present age of parents or - if deceased - their age at death (the older they are => less likely they will get ALS after the proband)	alive late fifties	alive late sixties	alive around 80 years of age	alive around 70 years of age	father died at age 48 from aggressive ALS, autopsy confirmed. Mother alive over 80 years old
Family history	None for NMD.<br>Both parents DM2	None for ALS or FTD; father DM. Nephew with Myotonic dystrophy	None for NMD or FTD	None for NMD.<br>both parents DM	Father with autopsy-confirmed ALS. No other affected family members
Unaffected siblings?	1 younger (not tested)	2 younger siblings (not tested)	2 younger brothers, not mutation carriers	8 older siblings, none are mutation carriers	1 sibling, not mutation carrier
Clinical diagnosis	sALS	sALS	sALS	sALS	fALS
Age at onset of first paresis	22 years	45 years	42 years	30 years	38 years
Site of first paresis	leg	right leg	left shoulder	left arm and hand	gluteal muscles and leg
Symptoms prior to onset of paresis	troublesome muscle cramps appeared with the onset of paresis and have persisted ever since	4 years before onset of paresis, severe muscle cramps and a tingling sensation in the right leg. These symptoms disappeared a year before onset of paresis	preparetic pain syndrome in the shoulder area	preparetic neuralgic pain initially in the shoulder region, later a period of pain sensation preceding manifest paresis in the legs	None
UMN/LMN affection	both	both	both	predominantly LMN	predominantly LMN
Survival time (paresis to death)	alive > 97 months after onset	alive > 41 months after onset	50,3 months	alive > 38 months	27,5
Total lifespan	alive, > 31 years	alive, >49 years	deceased at age 46 years	alive, > 33 years	41,1
Other genes excluded	large panel of neuromuscular-genes	NGS-WES; panel of ALS genes	ProjectMinE NGS-WGS; panel of ALS genes	NGS-WES	panel of ALS genes
Comments	No bulbar involvement. Participate in VALOR *tofersen* clinical drug trial	Edaravone treated	Late bulbar involvement	On invasive ventilation 16 months after onset	Late bulbar involvement
Disease rate	slow progression	slow progression	moderate progression	rapid progression	moderate progression
SOD1 enzymatic activity in erytrocytes, U/mg Hb (normal 54,1±5,7, ref. 11)	38,50	na	35,61	34,73	25,78
ACMG score (ref. 12)	PS1, PS2, PS4, PM1, PM2, PP3	PS2, PM2, PS4, PP3	PS2, PS4, PM2, PM5	PS2, PM1, PM2, PS4, PP3	PS1, PS4, PM1, PM2, PP3

DM, diabetes mellitus type 2; LMN, lower motor neuron signs; MND, motor neuron disease; NGS, next generation DNA sequencing; NMD, neuromuscular disease; WGS, whole-genome sequencing; WES, whole-exome sequencing;
