# Supplementary material for: De novo mutations in SOD1 are a cause of ALS
Source: J Neurol Neurosurg Psychiatry. 2021 Sep 13;93(2):201–6. doi: 10.1136/jnnp-2021-327520 (PMC8784989; doi:10.1136/jnnp-2021-327520)

Supplement 1a:

DNA sequencing results for the German family: the patient is heterozygous for *SOD1*:c.112G>C, p.Gly38Arg but not the parents.

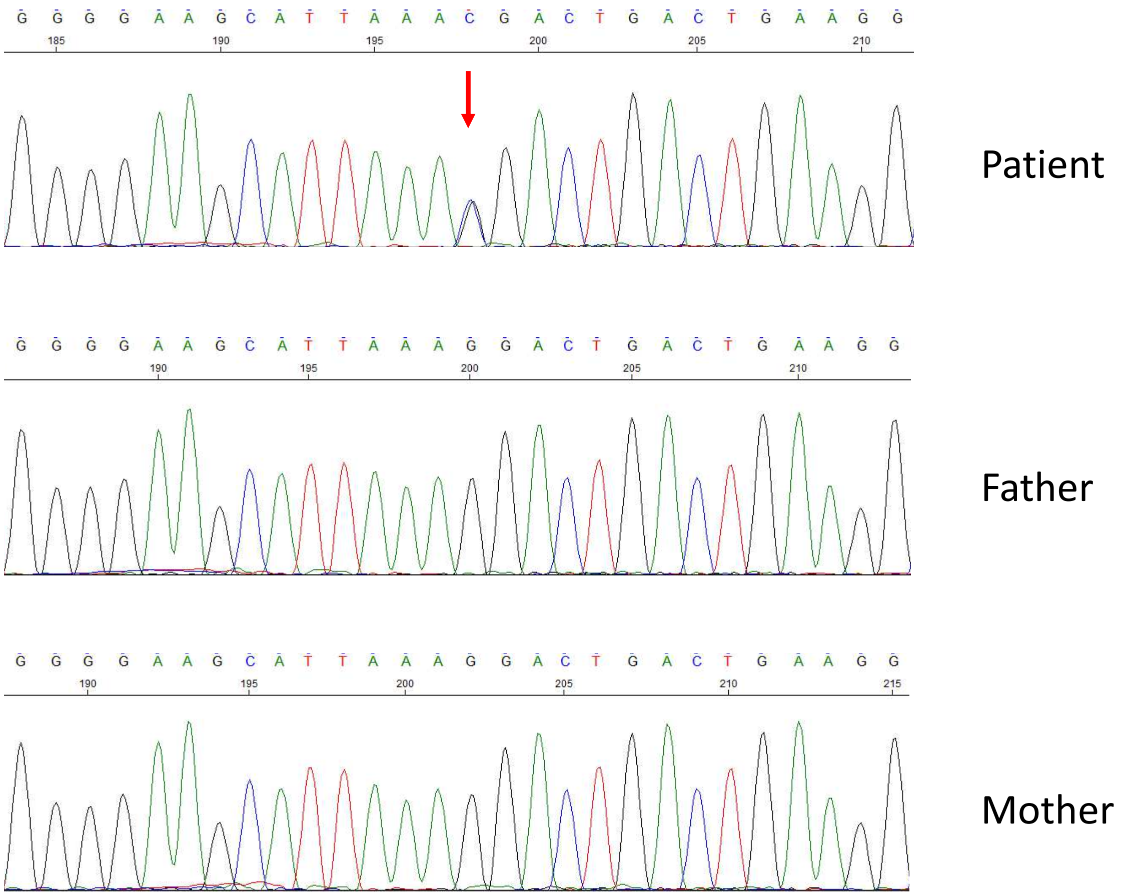

Supplement 1b South Korean family

DNA sequencing results for the Korean family: the patient is heterozygous for *SOD1*:c.268G>A, p.Ala90Thr but not the parents.

SOD1, NM_000454.4:c.268G>A (p.Ala90Thr)

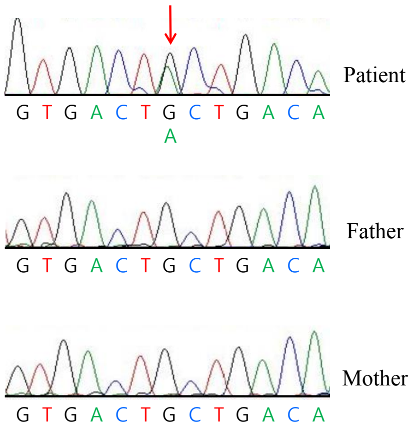

Supplement 1c Swedish family

DNA sequencing results for the Swedish family: the patient is heterozygous for *SOD1*:c.272A>T, p.Asp91Val but not the parents.

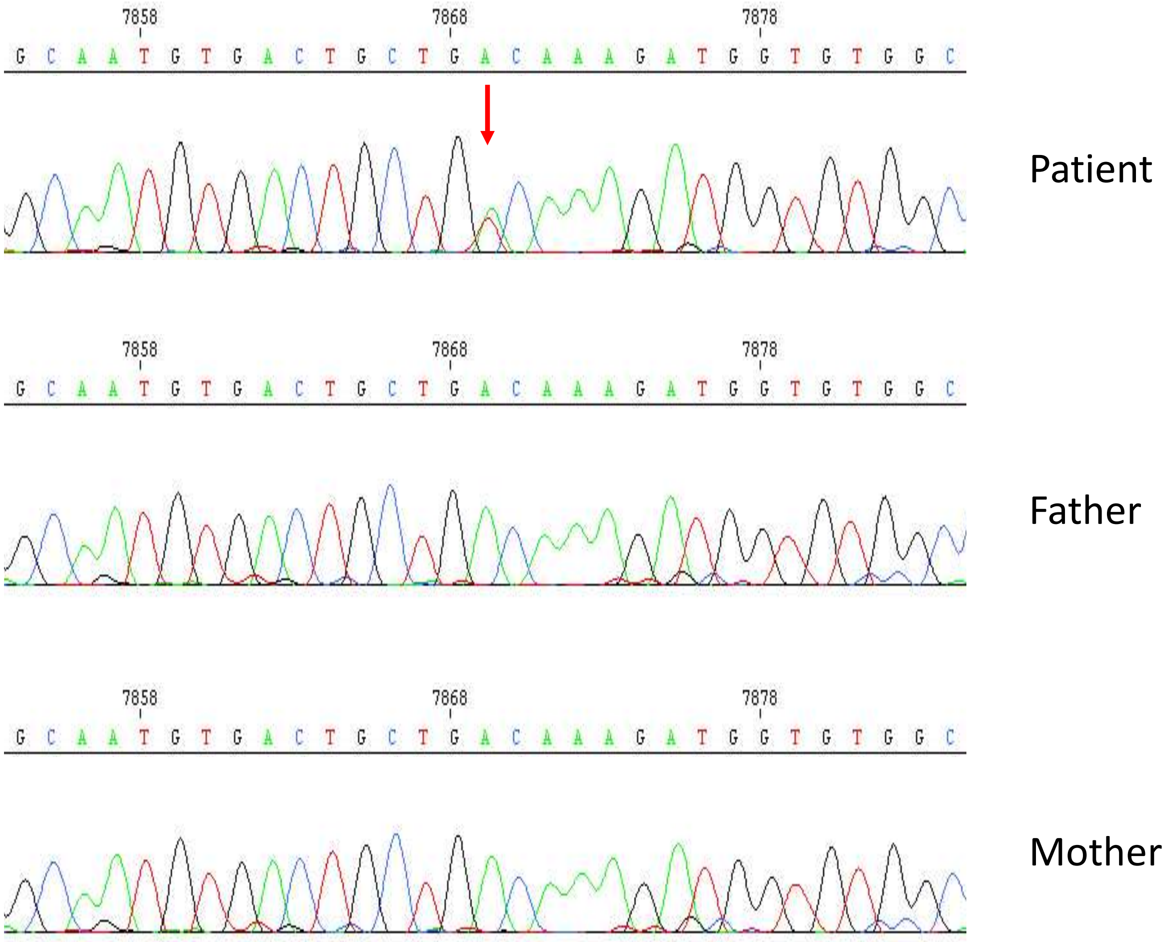

Supplement 1d

DNA sequencing results for the Kurdish-Turkish family: the patient is heterozygous for *SOD1*:c.304G>A, p.Asp102Asn but not the parents.

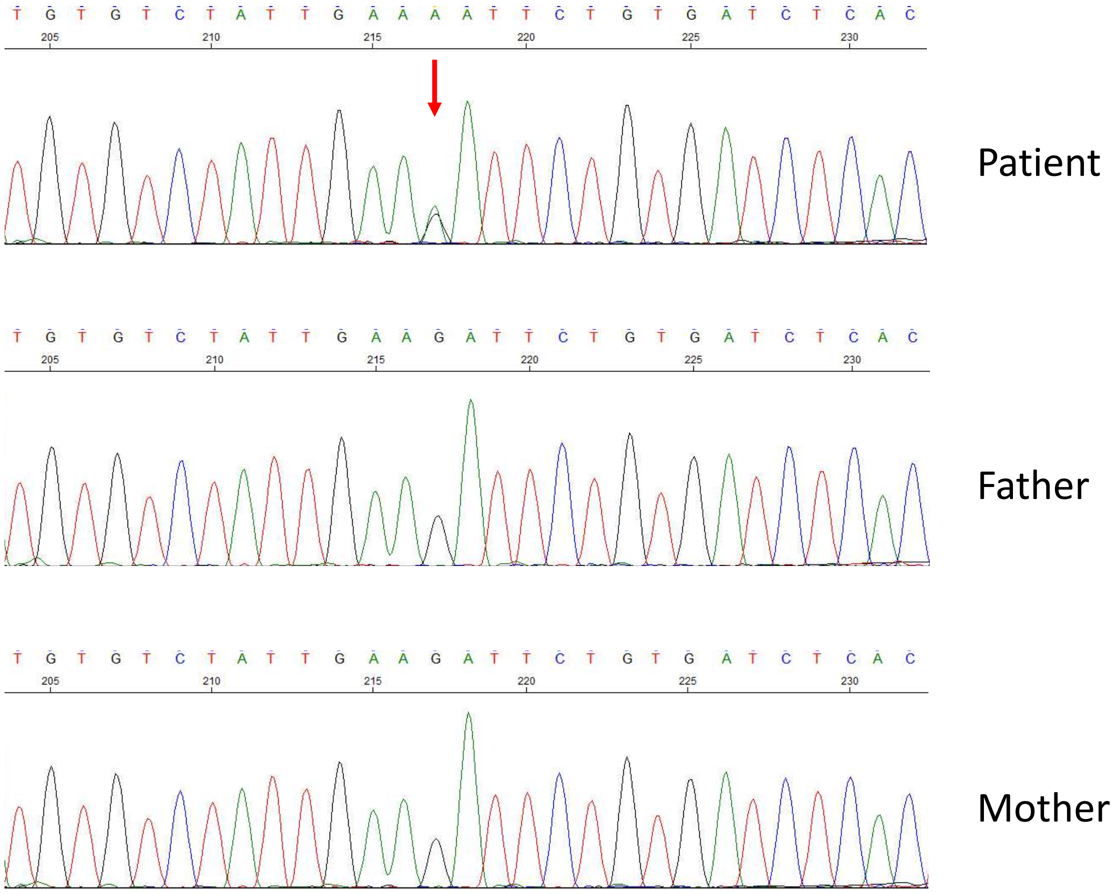

Supplement: Supplementary data [file jnnp-2021-327520supp002.pdf]
